# Patient satisfaction and associated factors in Addis Ababa’s public referral hospitals: insights from 2023

**DOI:** 10.3389/fmed.2024.1456566

**Published:** 2024-10-31

**Authors:** Dawit Abebe, Sinetibeb Mesfin, Luna Abebe Kenea, Yosef Alemayehu, Kostr Andarge, Temsegen Aleme

**Affiliations:** ^1^School of Nursing and Midwifery, College of Health and Medical Sciences, Jigjiga University, Jigjiga, Ethiopia; ^2^School of Nursing and Midwifery, College of Health and Medical Sciences, Haramaya University, Harar, Ethiopia; ^3^School of Medicine, Institute of Health, Jigjiga University, Jigjiga, Ethiopia; ^4^St. Peter’s Specialized Hospital, Addia Ababa, Ethiopia; ^5^Department of Epidemiology, School of Public Health, College of Health and Medical Sciences, Dilla University, Dilla, Ethiopia

**Keywords:** patient satisfaction, nursing care, inpatients, health care professionals, quality improvement

## Abstract

**Background:**

Currently, patient satisfaction is a major concern in the healthcare system of Ethiopia. Patient satisfaction with nursing care is considered an important factor in explaining patients’ service quality. Satisfied patients are more likely to have a good relationship with nurses, which suggests improved quality of care.

**Objective:**

To assess the prevalence of patient satisfaction and its associated factors among admitted patients in Addis Ababa city public referral hospitals, Ethiopia 2023.

**Methods and materials:**

An institutional-based cross-sectional study design was employed among 471 randomly selected patients from June 1 to July 30, 2023. Interviewer-administered a structured questionnaire was used to collect data. Patient satisfaction was measured by using the Newcastle Nursing Care Satisfaction Scale. Bivariable and multivariable logistic regressions were used to identify the factors associated with patient satisfaction.

**Result:**

471 participants responded among 506 selected patients yielding a response rate of 93%. The satisfaction of patients was 47.8% (95% CI = 42.9, 52.2%) Multiple logistic regression showed that participants aged 26–35 were less likely to be satisfied with nursing care [AOR = 0.25, 95% CI: 0.11, 0.56]. In contrast, those in the surgical ward [AOR = 3.85, 95% CI: 1.98, 7.45] and ophthalmology ward [AOR = 4.27, 95% CI: 1.81, 10.05] were more satisfied. No previous admission [AOR = 0.13, 95% CI: 0.07, 0.26], having no comorbidities [AOR = 13.4, 95% CI: 7.06, 25.4], and shorter admission duration [AOR = 9.14, 95% CI: 3.46, 24.11] were found to be factors with a significant association with patient nursing satisfaction.

**Conclusion:**

Overall, just under one in every two admitted patients were satisfied, indicating areas for potential improvement in nursing care. Specific factors such as patients in the age range of 26 to 35 reported significantly lower levels of satisfaction, whereas patients in the surgical and ophthalmology wards, as well as those without comorbidities and with shorter hospital stays, reported significantly higher levels of satisfaction. These findings emphasize the importance of targeted strategies to enhance nursing care.

## Background

Patient satisfaction is defined as the patient’s judgment on the quality of care in all aspects, particularly concerning the interpersonal process. Based on Andrea Eisenberg, 2020 patient satisfaction defined as a measure of how happy a patient is with their healthcare (1, 2). It is related to technical and interpersonal behavior, partnership building, immediate and positive nonverbal behavior, more social observation, courtesy, consideration, clear communication and information provision, respectful treatment, frequency of contact, length of consultation, service availability, and waiting time (3, 4).

Nurses and nursing care are very pertinent components of any healthcare system. It is a multidimensional concept that has the following aspects: the art of care, the technical quality of care convenience, cost, physical and environmental organization, availability of the resource, continuity of care, and outcomes (5, 6). Nursing symbolizes the art of care. Nurses provide emotional support, comfort, and advocacy for patients during often vulnerable times. Their ability to listen attentively, build rapport, and explain complex medical information clearly and compassionately is crucial for patient well-being (7). Additionally, nursing care is grounded in the technical quality of healthcare. Nurses are skilled professionals with a wide range of knowledge. They conduct thorough health assessments, create personalized care plans, perform nursing procedures, and provide patient education (8, 9).

Patients can assess the healthcare practitioners and services from their subjective perspective, even while they are unable to determine certain technical features. They are the best source of accurate information regarding clarity of explanations, the kindness of information they are receiving, barriers to obtaining care, or the nurse’s interpersonal behavior (10, 11). Patient satisfaction has been used as a significant indicator of quality services provided by healthcare personnel (9). As a result, patients’ satisfaction with nursing care is a significant factor influencing their overall satisfaction with health facility services (12–14). In Ethiopia, despite the Ethiopian Federal Ministry of Health develop various national quality management guidelines to enhance patient satisfaction, studies indicate that significant challenges persist in effectively implementing these guidelines (15, 16). A systematic review by Mulugeta et al. analyzed 15 studies and revealed a pooled prevalence of patient satisfaction with nursing care in Ethiopia was 55.15% (5).

Patient satisfaction with nursing care is related to the quality of nursing care (5). By exploring the levels of patient satisfaction, we can get insights into various aspects of the nursing services they receive. This feedback can highlight positive areas such as effective communication, empathy, and responsiveness, as well as areas needing improvement in overall patient engagement. Furthermore, by analyzing patient satisfaction, healthcare facilities can identify patterns and trends that inform quality improvement initiatives.

Even though this topic has been the subject of several studies in Ethiopia as well as globally, the results are still inconclusive and contradictory (17). Specifically in Ethiopia, almost all studies are limited to single health facilities that are set in the countryside (15, 18–20). The Ethiopian civil service reform strategy has been implemented in all hospitals to enhance service provision for the community (21). Based on this fact, it is crucial to understand the satisfaction levels of patients admitted to wards with more health facilities in Addis Ababa, where patients come from diverse regions using standard measurement tools to ensure reliable data. Additionally, a limited study was conducted on patient satisfaction with nursing care services in the country’s largest referral hospitals, which serve patients from a variety of regions. Therefore, this study is intended to assess the level of patient satisfaction and associated factors toward nursing care in Public Referral Hospitals of Addis Ababa, Ethiopia.

## Materials and methods

This study was conducted following principles outlined in the **Strengthening the Reporting of Observational Studies in Epidemiology (STROBE)** guidelines (22), ensuring rigor, transparency, and completeness in the reporting of our methodology and findings.

### Study design, population, and sample

An institutional-based cross-sectional study was conducted from June 1 to July 30, 2023, in two randomly selected public referral hospitals in Addis Ababa. The study population consisted of all admitted adult patients in these hospitals who met the inclusion criteria and provided consent to participate.

The sample size was determined using a single population formula, based on the assumption that the prevalence of patient nursing satisfaction is 49.2%, as reported in a study conducted at Debre Berhan Referral Hospital in Ethiopia (19). A 95% confidence level and a 5% margin of error (d) were applied. After accounting for a 10% non-response rate and a design effect of 1.5, the final sample size was calculated to be 506.

### Inclusion and exclusion criteria

All adult patients admitted for more than 24 h at the selected hospitals during the study period were included in the study. Patients who were seriously ill or unconscious at the time of data collection period were excluded from the study.

### Instruments and operational definition of variables

The Newcastle Nursing Care Satisfaction Scale (NSNS) was used to measure patient satisfaction. The satisfaction subscale assesses satisfaction with all aspects of nursing care using 19 items rated on a 5-point Likert scale (Not at All Satisfied, Barely Satisfied, Quite Satisfied, Very Satisfied, Completely Satisfied). The NSNS tool demonstrated excellent reliability with a Cronbach’s *α* of 0.96 from previous studies. A satisfaction sum score for each patient was calculated, and the mean score was used to dichotomize satisfaction into satisfied and unsatisfied. Based on this, the following definitions were made:

**Satisfied:** The mean score of the patient’s Newcastle Nursing Care Satisfaction Scale was 38.32. Respondents who scored above the mean value were regarded as satisfied with nursing practices.
**Unsatisfied:** Patients who scored below the mean on the Newcastle Nursing Care Satisfaction Scale were regarded as unsatisfied with nursing practices.

### Sampling procedure

Multistage sampling was used to get the study participants. The first two public referral hospitals were selected randomly after which the sample size (506) was allocated by probability proportional to size (PPS) considering their last one-month admission coverage before data collection. Again, this number was reallocated proportionally to the different wards of the selected hospitals excluding pediatric and neonatal admission wards. Systematic random sampling was employed to select the study participants from their sequence of admission registration during the study period. Every two admitted patients were considered for participation and the first patient was selected randomly at the beginning of the study. The sampling interval (K) was calculated by dividing the total sample size by the allocated sample size for each hospital. Similar approaches were applied to get the study participants in the selected wards.

### Data collection tools and procedure

#### Data collection instrument

Data were acquired through a structured, pre-tested, and interviewer-administered questionnaire which was developed by reviewing different literature. These carefully designed questionnaires encompass various sections, with **Part I:** focusing on the socio-demographic variables of the participant. **Part II**: Questions contained admission characteristics of the patient, and **Part III**: Questions to assess patients’ satisfaction toward nursing care with the Newcastle Nursing Care Satisfaction Scale (NSNS).

#### Data collection procedure

For data collection, BSc nursing staff were selected and trained to administer the questions. A private room at the hospital was used to interview patients to provide privacy and aid in the attainment of honest responses from the participants.

To supervise both the data collectors and the entire data collection process, two public health experts were selected. The principal investigator dedicated a day to providing training for the supervisor and data collectors, covering the study’s objectives, the content of the instruments, the participant selection process, how to fill out the questionnaire, and how to approach individuals ethically. All study participants had their understanding of the study’s aim, the consent form, the confidentiality issue, and informed consent guaranteed.

#### Data quality control

A half-day training session was conducted for both data collectors and supervisors. The questionnaire underwent a pre-testing phase conducted in Black Lion Hospital involving 25 patients that was a representative of 5% sample size. More importantly, the satisfaction scale was standard and used internationally by different scales. The questionnaire was prepared in the English language initially and translated into Amharic and then back to English to check their consistency by a bilingual language expert. Necessary modification in the questionnaire was made based on the nature of the gaps identified during the pretest. Data entered Epi-data version 7.2.1, cleaned and explored for outliers, missed values, and any inconsistencies.

#### Data processing and analysis

The collected data was cleaned and checked for completeness and consistency after which it was entered into Epi-data version 7.2.1. Then it was exported to Statistical Package for Social Science [SPSS] Version-27 software for analysis. Data exploration was done using, frequencies, percentages, and graphs. Cross-tabulation was done and variables that did not violate assumptions of logistic regression were entered into binary analysis. Variables with significant association in binary logistic regression with a *p*-value less than 0.25 were entered into multiple logistic analyses to control possible confounding variables and to identify independent predictor variables.

A multicollinearity test was conducted to assess the correlation between independent variables using the standard error and variance inflation factors. A multiple logistic regression model was used to declare the prediction of satisfaction by independent variables at a *p*-value less than 0.05 and Adjusted Odds Ratio (AOR) with 95% Confidence Interval (CI). The fitness was tested with Hosmer and Lemeshow significance values with variability explanations of satisfaction among satisfied and non-satisfied.

## Results

### Socio-demographic characteristics of respondents

**Figure 1:**
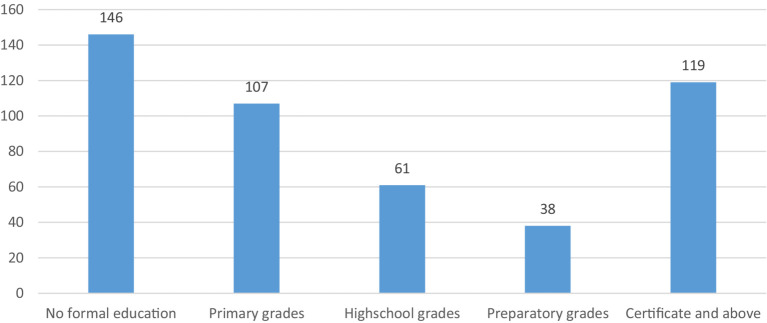
Educational status of patients admitted at public health hospitals of Addis Ababa, Ethiopia, 2023 (*N* = 471).

A total of 471 patients participated in the study with a response rate of 93%. The mean (±SD) age of the participants was 37.76 (±12.17) years. Based on the findings (33.8%), (51.8%), (71.5%), and (63.9%) were between the age group 26–35 years, female, married, and urban residents, respectively (Table 1). Regarding educational status; 146 (30.9%) participants had no formal education while 119 (25.2%) had certificate and above grades (Figure 1).

**Table 1:** Socio-demographic characteristics of admitted patients in public health hospitals of Addis Ababa, Ethiopia, 2023 (*n* = 471).

Variables	Frequency	Percent
Age (completed years)		
18–25	59	12.5
26–35	159	33.8
36–45	125	26.5
46–55	96	20.4
56+	32	6.8
Sex		
Male	227	48.2
Female	244	51.8
Marital status		
Currently married	337	71.5
single	106	22.5
Others/Widowed/Divorced	28	5.9
Ethnicity		
Amhara	115	24.4
Oromo	245	52.0
Gurage	20	4.2
Tigre	28	5.9
Others	63	13.4
Current residence		
Urban	301	63.9
Rural	170	36.1
Have family support during admission		
Yes	427	90.7
No	44	9.3
Occupation of patient		
Housewife	150	31.8
Government employee	18	3.8
Merchant	53	11.3
Farmer	40	8.5
Private/self-employee	148	31.4

### Admission characteristics of patients

The average length of stay for patients, indicated by the mean (±SD), was 9.2 (±9.7) days. The majority (43.5%) had stayed in the hospital for 4–7 days, with the next most common duration being 8–14 days. Additionally, a significant portion of participants were from the standard admission rooms (Table 2).

**Table 2:** Admission characteristics of patients admitted at public health hospitals of Addis Ababa, Ethiopia, 2023.

Variables	Number	Percent
Admission ward		
Medical	122	25.9
Surgical	123	26.1
Gynecology/obstetrics	69	14.6
Ophthalmology	97	20.6
Orthopedics	60	12.7
Admission rooms		
Usual	407	86.4
Unusual/Isolated rooms	64	13.6
Marital status		
Currently married	337	71.5
Single	106	22.5
Others/Widowed/Divorced	28	5.9
History of previous admission		
Yes	208	44.2
No	263	55.9
Presence of comorbid disease		
Yes	232	49.3
No	239	50.7

### Newcastle nursing care satisfaction scale scores of patients

In this study, participants were asked to rate their satisfaction using the Newcastle Nursing Care Satisfaction Scale (NNCSS). The results showed that a significant proportion of patients were content with the time nurses spent interacting with them, with 41.4% being quite satisfied and 14.2% very satisfied. However, only 27.0% were very satisfied with the responses from nurses. Additionally, 41.6% of patients reported being barely satisfied with the frequency of nurses checking on their well-being. Concerning patient privacy, only 32.1% of patients were completely satisfied with the privacy provisions during their care (Table 3).

**Table 3:** Satisfaction of patients admitted at public health hospitals of Addis Ababa based on Newcastle nursing care satisfaction scales (NNCSS) (*N* = 471).

Newcastle nursing care satisfaction scale questions	Possible answers [frequency (%)]				
	Not at all satisfied	Barely satisfied	Quite satisfied	Very satisfied	Completely satisfied
Amount of time nurses spent with you	9 (1.9)	185 (39.3)	195 (41.4)	67 (14.2)	15 (3.2)
Capability of nurses at their job	7 (1.5)	175 (37.2)	121 (25.7)	158 (33.5)	10 (2.1)
Nurses’ knowledge of patient care	10 (2.1)	121 (25.7)	177 (37.6)	134 (28.5)	29 (6.2)
How quick are nurses	4 (8.0)	165 (35.0)	113 (24.0)	127 (27.0)	62 (13.2)
Nurses made you feel at home	36 (7.6)	110 (23.4)	171 (36.3)	94 (20.0)	60 (12.7)
The amount of information nurses gives to you about your condition	33 (7.0)	184 (39.1)	123 (26.1)	71 (15.1)	60 (12.7)
A nurse around when you need	36 (7.6)	153 (32.5)	178 (37.8)	62 (13.2)	42 (8.9)
How often do nurses check to see you if you are well	24 (5.1)	196 (41.6)	136 (28.9)	105 (22.3)	10 (2.1)
Nurses’ helpfulness	7 (1.5)	179 (38.0)	176 (37.4)	91 (19.3)	18 (3.8)
Nurses explaining things	36 (7.6)	227 (48.2)	108 (22.9)	72 (15.3)	28 (5.9)
Nurses help put your relatives’ minds at rest	24 (5.1)	182 (38.6)	110 (23.4)	130 (27.6)	25 (5.3)
Nurses’ manner in doing their work	33 (7)	98 (20.8)	155 (32.9)	123 (26.1)	62 (13.2)
The type of information nurses gave to you about your condition and treatment	53 (11.3)	188 (39.9)	140 (29.7)	38 (8.1)	52 (11)
Treatment of a patient as an Individual	36 (7.6)	109 (23.1)	118 (25.1)	172 (36.5)	36 (7.6)
Listening to patient worries/concerns	33 (7.0)	127 (27.0)	143 (30.4)	138 (29.3)	30 (6.4)
Amount of freedom provided at the ward	9 (1.9)	133 (28.2)	159 (33.8)	143 (30.4)	27 (5.7)
Nurses willing to respond to requests	32 (6.8)	146 (31.0)	133 (28.2)	134 (28.5)	26 (5.5)
Amount of privacy nurses provided	47 (10)	133 (28.2)	83 (17.6)	57 (12.1)	151 (32.1)
Nurses’ awareness of needs	32 (6.8%)	185 (39.3)	59 (12.5)	93 (19.7)	102 (21.7)

### Prevalence of patient satisfaction

**Figure 2:**
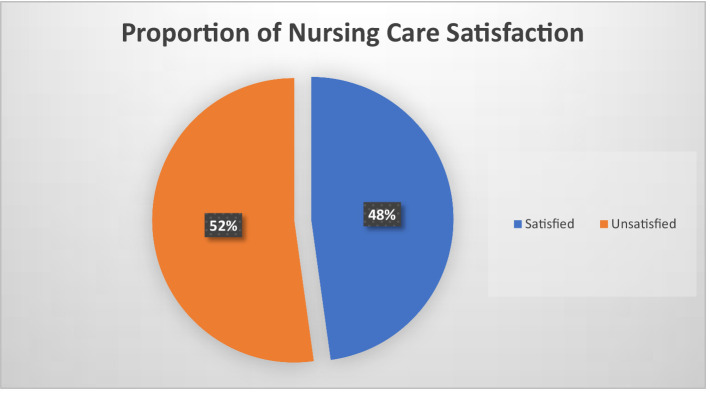
Proportion of nursing care satisfaction among patients admitted at public health hospitals of Addis Ababa, Ethiopia, 2023.

The average score of patient satisfaction, represented by the mean (±SD), was determined to be 36.32 (± 7.4). Following the operational definition outlined in the research, patients were categorized as unsatisfied if they scored below the mean and satisfied if they scored equal to or above it. This classification resulted in 47.8% (95% CI = 42.9, 52.2%) of patients being classified as satisfied (Figure 2).

### Factors associated with patient satisfaction

The comparison between satisfied and unsatisfied patients revealed distinct factor variables linked to patient satisfaction. Initially, twelve variables, encompassing socio-demographic and admission characteristics, were presumed to be associated with patient satisfaction. Through bivariate analysis, six of these variables demonstrated associations with a *p*-value less than 0.25, prompting their inclusion in multivariable analysis. In the model, participant in the age group of 26–35 [COR = 0.40 (95% CI: (0.21, 0.73))], 36–45 [COR = 1.96 (95% CI: (1.028, 3.76))], ruler residence [COR = 1.74 (95% CI: (1.188, 2.55))], participant admitted to surgical ward [COR = 3.54 (95% CI: (2.09, 6.0))], obstetrics and gynecology ward [COR = 3.07 (95% CI: (1.66, 5.66))], and patient admitted to ophthalmology ward [COR = 6.0 (95% CI: (3.31, 10.89))], having no history of previous admission [COR =  0.34 (95% CI: (0.23, 0.50))], having no comorbidity [COR =  4.96 (95% CI: (3.35, 7.33))] were found to have a statistically significant positive association with patient nursing care satisfaction.

In the multivariate analysis, participants in the age group of 26–35 were less likely to be satisfied with nursing care [AOR = 0.25, 95% CI: 0.11, 0.56]. Conversely, those admitted to the surgical and orthopedic wards were almost four times more likely to be satisfied with nursing care [AOR = 3.85, 95% CI: 1.98, 7.45]. Additionally, participants within the ophthalmology ward were also around four times more likely to be satisfied with nursing care [AOR = 4.27, 95% CI: 1.81, 10.05].

Having no history of previous admission was associated with lower satisfaction at [AOR = 0.13, 95% CI: 0.07, 0.26] while having comorbidity was strongly associated with higher satisfaction [AOR = 13.4, 95% CI: 7.06, 25.4]. Moreover, the duration of admission also played a significant role in patient satisfaction [AOR = 9.14, 95% CI: 3.46, 24.11]. These factors maintained their significant associations with patient nursing satisfaction in the multivariable analysis (Table 4).

**Table 4:** Factors associated with nursing care satisfaction among patients admitted at public health hospitals of Addis Ababa, Ethiopia, 2023.

Variables category	Nursing care satisfaction				Bivariate logistic regression		Multivariate logistic regression
	Satisfied		Unsatisfied				
	N	%	N	%	COR	*p*-value	AOR (95% CI)
Age category							
18–25	25	42.4	34	57.6	1		1
26–35	103	64.8	56	35.2	0.40	0.003	0.25 (0.11, 0.56) **
36–45	34	27.2	91	72.8	1.96	0.041	0.93 (0.35,2.49)
46–55	45	46.9	51	53.1	0.83	0.585	0.90 (0.37, 2.15)
56+	17	53.1	15	47.9	0.64	0.327	0.38 (0.10, 1.43)
Current residence							
Urban	158	52.5	143	47.5	1		1
Rural	66	38.8	104	61.2	1.74	0.004	1.69 (0.997, 2.86)
Admission wards							
Medical	81	66.4	41	33.6	1		1
Surgical	44	35.8	79	64.2	3.54	0.000	3.85 (1.98, 7.45)**
Oby/Gyne	27	39.1	42	60.9	3.07	0.000	1.95 (0.87, 4.35)
Ophthalmology	24	24.7	73	75.3	6.0	0.000	4.27 (1.81, 10.05)*
Orthopedics	48	80	12	20	0.49	0.060	0.67 (0.27, 1.69)
History of previous admission							
Yes	69	33.2	139	66.8	1		1
No	155	58.9	108	41.1	0.32	0.000	0.13 (0.07, 0.26)**
Comorbid disease							
Yes	155	66.8	77	33.2	1		**1**
No	69	28.9	170	71.1	4.96	0.000	13.4 (7.06, 25.4)**
Length of stay in hospital							
< = 3 days	69	44.2	87	55.8	0.66	0.205	9.14 (3.46, 24.11)**
4–7 days	87	54.4	73	45.6	1.05	0.072	0.969 (0.40, 2.30)
8–14 days	39	42.9	52	57.1	0.95	0.834	0.565 (0.23, 1.34)
> = 15 days	29	45.3	35	54.7	1		1

* = *p*-value <0.05, ** = *p*-value <0.005. CI, confidence interval; COR, crude odds ratio; AOR, adjusted odds ratio. The bold text indicates values that are significantly associated with the outcome variable.

## Discussion

Patients’ satisfaction has been used as a significant indicator of quality services provided by health care providers. Consequently, the most important predictor of patients’ overall satisfaction with hospital care is particularly related to their satisfaction with nursing care. The present study aimed to assess the prevalence of patient satisfaction and its associated factors among patients admitted to public hospitals in Addis Ababa city.

The study showed 47.8% of patients were satisfied which means just under one in every two admitted patients were satisfied. The prevalence is almost consistent with the findings in a study conducted at Debre Berhan Hospital that reported 49.2% patient satisfaction (18) and studies done in eastern Ethiopia (52.75%) (23), and the pooled prevalence (55.15%) of patient satisfaction in Ethiopia (5). It is also almost consistent with the study conducted in Menelik II Hospital in 2016 which reported 54.8% patient satisfaction (24).

However, the prevalence was slightly lower than the studies conducted in Dessie Ethiopia, Mizan-Aman, Bonga and Tepi hospitals and Pawie General Hospital, which reported 58.8, 61.3, and 60.8%, respectively (25–27). It is also lower when it compared to a study in Iran that reported 69% satisfaction (28). The difference could be attributed to differences in the socioeconomic status of study participants in different areas. In the case of Iran, the discrepancy might be due to different standards or procedures of patient care. Additionally, this discrepancy may arise from differences in how satisfaction is operationalized and the techniques used to determine satisfaction status. In this study, patient satisfaction was defined using the mean score as a cut-off point.

Several independent variables were indicated to have a significant association with the dependent variable. Multivariate logistic regression showed that patients in the age group 26–35 were 75% less likely to be satisfied with nursing care. This might be possible because to patients aged 26–35 often is the age significant work and family responsibilities are apparent making hospital stays particularly disruptive. This disruption might contribute to lower satisfaction levels. In the present study patients who came from rural areas were more likely satisfied than those from urban areas. The finding is supported by the study done on patient satisfaction in urban and rural areas of Scotland (29). This might be the fact that most patients from rural areas might have lower expectations regarding healthcare services compared to those from urban areas. The other possible explanation is that most rural dweller in Ethiopia enrolled in community-based health insurance (CBHI) (30), so their healthcare expense is covered through these insurers.

Patients with no prior history of admission were about 87% less likely to be satisfied than those who used to have previous history of admission. The possible explanation might be that this group of patients could not have had experiences with admission that their expectations and the standards they found might affected their satisfaction. This is supported by the study conducted at Debre Berhan Hospital (18). Presence of comorbidity disease other than the present admitting case was also a factor that significantly affected patient satisfaction, patients who had no comorbidity were more likely to be satisfied than those who had comorbidity This could be because those patients with other diseases apart from the current health problem need extra nursing care and follow up while those without other comorbidities do not need extra nursing care that allows appropriate, timely and frequent follow up which increase patient satisfaction. This finding is supported by a study conducted at Dessie (25).

Patients admitted in surgical and ophthalmology wards were more likely to be satisfied than patients in others. The findings are in line with the study done at Pawie General Hospital, west Ethiopia, where patients in the surgical ward were about three times more likely to be satisfied (27). This might be due to surgical and ophthalmology procedures often having well-defined goals and measurable outcomes. This clear goal can lead to a sense of accomplishment for patients. The finding that patients with shorter stays (less than or equal to 3 days) reported higher satisfaction aligns with a study done in an Academic Hospital (31). One possible explanation is that patients with shorter stays may have experienced less severe health issues or undergone less invasive treatments, leading to quicker recovery and higher satisfaction levels.

### Strengths and limitations

The present study used a standard tool (NWNCSS) to precisely estimate patient satisfaction. The study adds to the knowledge of understanding of patient satisfaction in admitted patients by widening the study setting to two referral hospitals. Data collectors were nurse professionals who could explain the questions adequately which might have increased data quality. Recall biases are unlikely in the present study as information inquired were present experiences.

Regarding limitations, social desirability bias while participants were requested to give accurate information about services provided while receiving care might have influenced the result of the study. However, they were assured that the interview was for the improvement of services. Furthermore, one potential limitation is that the questionnaire was administered by an interviewer rather than through self-administration by the participants. We recommend that future researchers explore solutions to this limitation to enhance the validity of their findings and minimize bias.

## Conclusion

Overall, just under one in every two admitted patients were satisfied, indicating areas for potential improvement in nursing care. Specific factors with satisfaction include the age group 26–35 showing lower satisfaction levels, indicating a need for targeted strategies to address their specific needs. While patients in surgical and ophthalmology wards, along with those with no comorbidities and shorter admission durations, reported significantly higher satisfaction levels. These results highlight the importance of considering demographic and clinical factors when developing care strategies. By focusing on these elements, hospitals can improve the quality of nursing care and enhance overall patient satisfaction.

## Data Availability

The original contributions presented in the study are included in the article/supplementary material, further inquiries can be directed to the corresponding author.
